# Connective Tissue Growth Factor in Digestive System Cancers: A Review and Meta-Analysis

**DOI:** 10.1155/2020/8489093

**Published:** 2020-12-27

**Authors:** Feng Zhao, Chan Li, Yun Wu, Jianling Xia, Ming Zeng, Tao Li, Ke Xie

**Affiliations:** ^1^Oncology Clinical Department, Sichuan Provincial People's Hospital, University of Electronic Science and Technology of China, Chengdu 611731, China; ^2^Southwest Medical University, People's Hospital of Xinjin, Chengdu 611430, China; ^3^Medical School, University of Electronic Science and Tech, Chengdu 611731, China; ^4^Southwest Medical University, Sichuan Cancer Hospital and Institute, Chengdu 610041, China

## Abstract

**Aim:**

A meta-analysis was conducted to estimate the impact of connective tissue growth factor (CTGF) on outcomes in patients with digestive system cancers.

**Methods:**

A systemic literature survey was performed by searching the Cochrane Library and PubMed databases for articles that evaluated the impact of CTGF on outcomes in patients with digestive system cancers. Hazard ratios and 95% confidence intervals were calculated for prognostic factors, overall and recurrence-free survival using RevMan 5.3 software.

**Results:**

This meta-analysis was conducted to evaluate a total of 11 studies that included 1730 patients. The results showed that elevated CTGF expression was significantly correlated with advanced age, larger tumor size, multiple tumors, and vascular invasion. Subgroup analysis by cancer type revealed increased risk for lymph node metastasis and advanced tumor node metastasis (TNM) stage in gastric cancer, compared with colorectal cancer. An unfavorable effect of elevated CTGF levels on overall survival was found in patients with hepatocellular carcinoma and patients with gastric cancer, while survival was improved in colorectal cancer patients with high CTGF expression, compared to those with normal levels of CTGF.

**Conclusions:**

Elevated CTGF expression may be a novel biomarker for disease status and predicted survival outcomes in patients with specific digestive system cancers.

## 1. Introduction

Cancer of the digestive system is one of the leading causes of cancer-related death worldwide [1]. Hepatocellular carcinoma (HCC), gastric cancer (GC), and colorectal cancer (CRC) are associated with high rates of mortality and enormous economic burdens [2–4]. Although there have been great advances in surgical techniques and postoperative therapy, patients with these cancers have poor clinical prognosis due to advanced-stage disease or tumor metastasis [5–7]. Therefore, the discovery and identification of potential biomarkers are essential to find novel anticancer strategies.

Connective tissue growth factor (CTGF), known as CCN2, is a member of the CCN superfamily of glycoproteins [8]. The protein has been found to mediate the pathogenesis of organ fibrosis, cardiac atherosclerosis, and fibrotic skin disease [9–11]. In recent years, numerous studies have revealed that CTGF expression is also associated with cancer. Although previous studies have demonstrated the upregulation of CTGF in a range of human cancers with poor prognosis, some studies reported conflicting results [12–16]. The role that CTGF plays in the diagnosis and prognosis of digestive system cancer remains unknown. We designed this meta-analysis to evaluate the correlation between levels of CTGF and clinical characteristics and prognosis in patients with digestive system cancers.

## 2. Materials and Methods

### 2.1. Literature Search

A systematic literature search of the PubMed and Cochrane Library databases was performed. The search included any publication added to the database before December 21, 2019. The following keywords were included in the search: “(connective tissue growth factor OR CTGF OR CCN family member 2 (CCN2) OR hypertrophic chondrocyte-specific gene product 24 (Hcs24) OR insulin-like growth factor-binding protein 8 (IGFBP-8) and (diagnosis OR outcome OR prognosis OR prognostic factor OR survival) and (cancer OR carcinoma OR tumor OR malignancy OR digestive system cancer OR gastric cancer OR colorectal cancer OR gallbladder cancer OR hepatocellular carcinoma OR pancreatic cancer).”

### 2.2. Inclusion and Exclusion Criteria

The inclusion criteria for retrieved studies were as follows: (1) studies with confirmed pathological diagnosis of digestive system cancers, (2) stratification of patients based on high vs. low expression of CTGF, and (3) relevance of CTGF expression and clinical characteristics or survival outcomes. The exclusion criteria were as follows: (1) case reports, editorials, meta-analyses, reviews, and conference reports; (2) insufficient data; (3) studies conducted in animal models; and (4) duplications of published reports or articles.

### 2.3. Study Selection and Quality Assessment

Data were extracted from the studies included in the meta-analysis by two independent investigators. The following information was extracted: first author, year, country, types of cancer, sample size, detection method, number of patients with a change in the expression of CTGF, tumor size, lymph node metastasis, differentiation grade, tumor-node-metastasis (TNM) stage, tumor number, vascular invasion, and capsule formation. Hazard ratios (HRs) and corresponding 95% confidence intervals (CI) were carefully collected or calculated. The quality of involved studies was evaluated by independent investigators using the Newcastle-Ottawa scale (NOS) which consists of subject selection, comparability of study groups and measurement of survival outcomes [17]. Articles with NOS scores ≥ 6 were considered to be of high quality.

### 2.4. Statistical Analysis

The included data were analyzed using Review Manager version 5.3 (Revman, The Cochrane Collaboration, Oxford, UK). The correlation between levels of CTGF and clinical outcomes was assessed by calculating HRs and corresponding 95% CIs. Heterogeneity among studies was evaluated with Cochrane *Q* and Higgins *I*^2^ statistics. A fixed effects model was used when *I*^2^ < 50% and the corresponding *P* value < 0.05. For pooled results, a random effects model was used. The results obtained were analyzed using Review Manager version 5.3 with *P* < 0.05 considered to be statistically significant. Subgroup and sensitivity analyses were conducted to analyze the sources of heterogeneity.

## 3. Results

**Figure 1:**
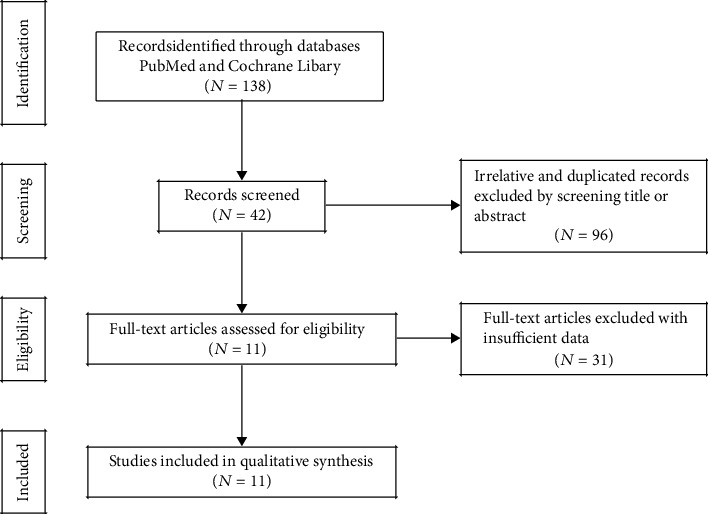
Flow diagram of article and study selection process.

The search strategy described above yielded 138 studies. We excluded 96 of these studies because of irrelevance or duplication. Another 31 studies were excluded because of insufficient data for a full-text review. Eleven studies, which included 12 cohort studies, ultimately met the meta-analysis inclusion and exclusion criteria (Figure 1). These studies were conducted between 2005 and 2018 and included 1730 patients in 4 countries [18–28].

The main characteristics of the included studies are presented in Table 1. Six types of digestive system cancer were evaluated, including 4 cohorts of HCC, 3 cohorts of GC, 2 cohorts of CRC, and 1 cohort each of intrahepatic cholangiocarcinoma (ICC), esophageal squamous cell carcinoma (ESCC), and gallbladder cancer (GBC). The main clinical parameters analyzed included age, gender, tumor size, lymph node metastasis, tumor differentiation, TNM stage, tumor number, vascular invasion, and capsule formation (Suppl. Table 1). In most studies, for tissue to be considered cancerous, >50% of cells had positive staining for immunohistochemical tumor markers. In addition, all included studies got NOS scores of 7 or more, demonstrating the high research quality.

### 3.1. Association between CTGF Expression and Clinical Characteristics

**Figure 2:**
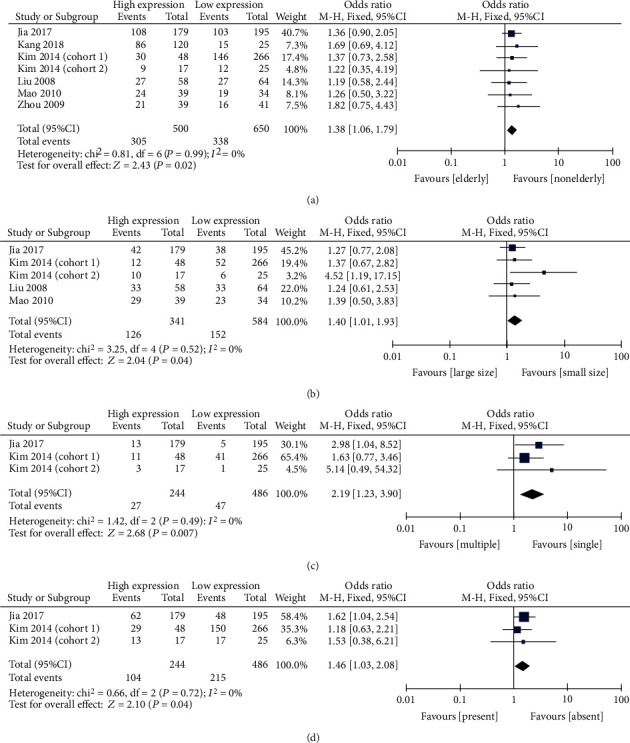
Meta-analysis of the relationship between elevated levels of CTGF and clinical characteristics: (a) age, (b) tumor size, (c) tumor number, and (d) vascular invasion.

**Figure 3:**
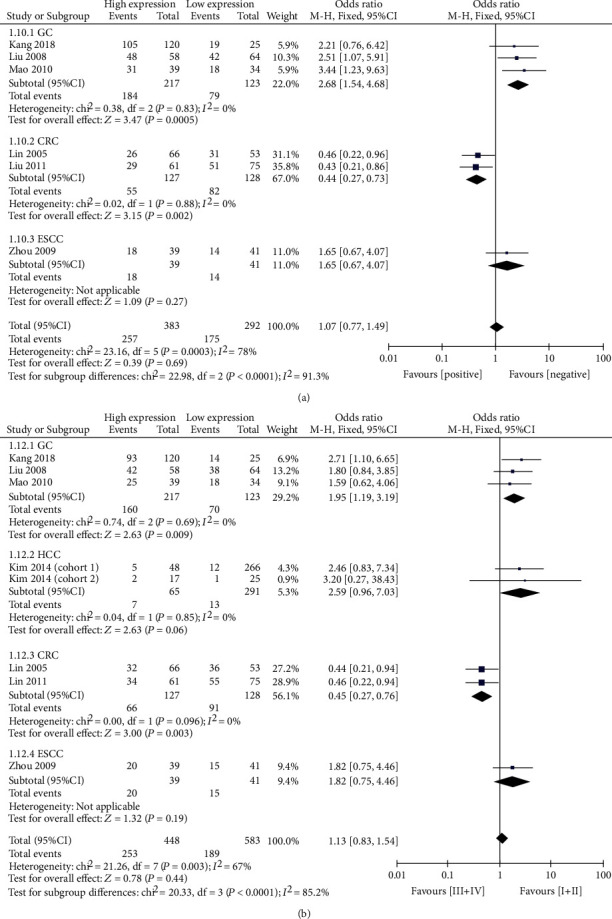
(a) Meta-analysis of the relationship between elevated levels of CTGF and lymph node metastasis, stratified by cancer type. (b) Meta-analysis of the relationship between elevated levels of CTGF and TNM stage, stratified by cancer type.

The pooled results demonstrate that elevated levels of CTGF were significantly associated with multiple tumors (OR = 2.19, 95% CI: 1.23-3.90), larger tumor size (OR = 1.40, 95% CI: 1.01-1.93), advanced age (OR = 1.38, 95% CI: 1.06-1.79), and vascular invasion (OR = 1.46, 95% CI: 1.03-2.08; Table 2, Figure 2). However, elevated levels of CTGF were not found to be significantly associated with gender, lymph node metastasis, tumor differentiation, TNM stage, or capsule formation (Suppl. Figure 1). Subgroup analysis for various types of cancer was conducted. The results showed that the overexpression of CTGF was associated with increased risk for lymph node metastasis (OR = 2.68, 95% CI: 1.54-4.68) and advanced TNM stage (OR = 1.95, 95% CI: 1.19-3.19) in GC. In CRC, elevated CTGF expression was associated with decreased risk for lymph node metastasis (OR = 0.44, 95% CI: 0.27-0.73) and lower TNM stage (OR = 0.45, 95% CI: 0.27-0.76) (Figure 3). CTGF expression was not associated with the stage of pathological differentiation in any type of cancer investigated (Suppl. Figure 2).

### 3.2. Association between CTGF Expression and Survival Outcomes

**Figure 4:**
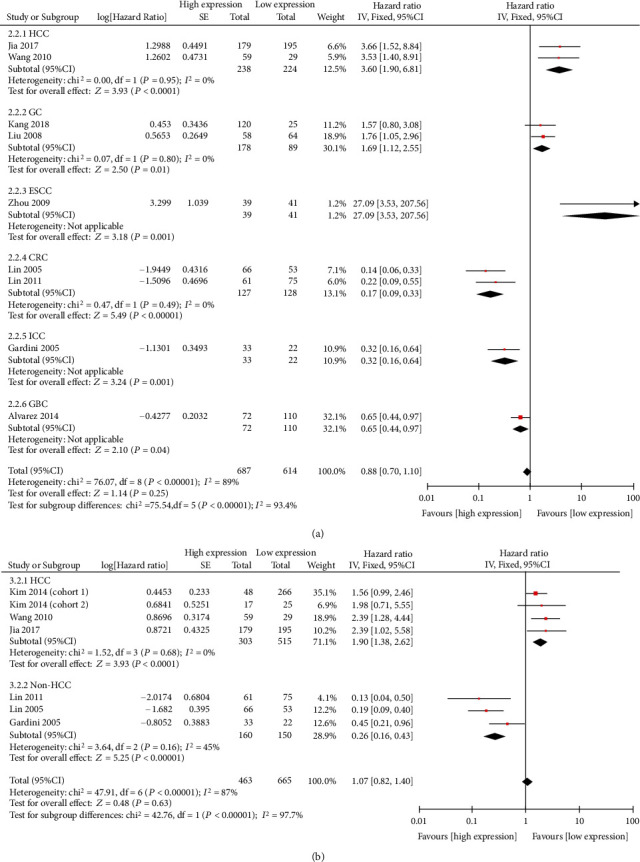
Meta-analysis of combined HRs for elevated levels of CTGF, with the results stratified by cancer type. Combined HRs for (a) OS and (b) DFS.

Nine studies, which included 1301 patients, assessed the correlation between CTGF expression and overall survival (OS). The pooled HR was 1.05 (95% CI: 0.50-2.23, *P* = 0.89), demonstrating no significant association between CTGF expression and patient outcomes (Suppl.  Figure 3a). The results of subgroup analysis by cancer type revealed that increased levels of CTGF were significantly associated with poor OS in patients with HCC (HR = 3.60, 95% CI: 1.90-6.81) and patients with GC (HR = 1.69, 95% CI: 1.12-2.55). In CRC, elevated CTGF expression was associated with improved survival outcomes (HR = 0.32, 95% CI: 0.16-0.64, fixed effects) (Table 3 and Figure 4(a)). When the data were stratified for sample size, technology, and region where the study was conducted, no significant association between CTGF and OS was found (Suppl.  Figure 5).

For disease-free survival (DFS), we performed analysis based on seven studies, which included 1128 patients. The results of pooled analysis showed that, similar to OS, DFS had no significant association with CTGF expression (Suppl.  Figure 3b). However, subgroup analysis based on cancer type revealed that elevated CTGF expression was a strong prognostic factor for poor DFS in patients with HCC (HR = 1.90, 95% CI: 1.38-2.62) (Figure 4(b)).

## 4. Discussion

**Figure 5:**
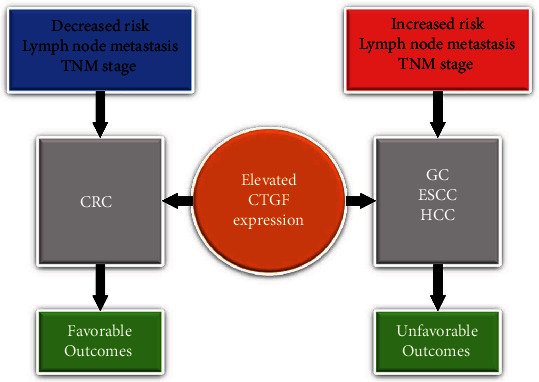
Demonstration of different roles CTGF played in specific digestive system cancers.

It has been more than 20 years since CTGF was first found to play a role in human cancer [29, 30]. To date, CTGF expression has been found to be associated with various tumor types, including HCC [31], pancreatic cancer [32], lung cancer [33], and prostate cancer [34]. The role that CTGF plays in digestive system cancers is diverse and tumor specific (Figure 5). We therefore conducted a meta-analysis to estimate the clinical value of CTGF in predicting outcomes for patients with digestive system cancers.

Our data indicated that elevated CTGF expression has a significantly unfavorable impact on OS and DFS in patients with HCC. HCC patients with high levels of CTGF were more likely to have multiple tumors and vascular invasion. Our results were in line with those of a previous study by Makino et al. [35]. The results of their study showed that elevated levels of CTGF expression were associated with increased risk for multiple tumors, portal invasion, and advanced tumor classification. The authors identified CTGF as a key modulator in the HCC microenvironment that activates the progrowth Ras/Mek/Erk signaling pathway to promote HCC growth. CTGF has also been found to promote angiogenesis in HCC after HBV infection or hypoxia [36, 37]. Thus, high expression of CTGF is regarded as a biomarker for worse survival outcomes in patients with HCC. The unfavorable effects on prognosis may be caused by the malignant characteristics induced by CTGF.

Research on patients with GC revealed a significant association between increased expression of CTGF and poor OS. Patients with high CTGF expression had increased risk for lymph node metastasis and advanced TNM stage. CTGF was overexpressed in GC compared to normal tissue. Increased CTGF promoted the transformation of cancer cells into peritoneal mesothelial cells through the epithelial to mesenchymal transition, which increased the mobility of cancer cells, facilitating migration and invasion [38]. Activation of the *α*5*β*3/ERK1/2 pathway may be responsible for this increase in cell motility [39]. However, high levels of CTGF were associated with different outcomes in patients with CRC. The different roles played by CTGF in GC and CRC may reflect diverse molecular mechanisms. In *ex vivo* studies of CRC, CTGF was shown to decrease the ability of CRC cells to adhere to the peritoneum. This effect was also mediated by the activation of integrin *α*5 [25]. In terms of other digestive system cancers, elevated CTGF expression is associated with worse survival in ESCC and with improved outcomes in ICC and GBC. Due to the limited number of studies on these topics, we could not accurately determine the function of CTGF in these cancers. More studies and further analysis are necessary.

This meta-analysis had some limitations. Firstly, our study only included a total of 11 studies, which investigated 6 types of digestive system cancer. Because various types of cancer were included in the meta-analysis, the pooled results presented heterogeneity. The limited availability of studies on specific cancers prevented us from evaluating publication bias. Second, the cut-off value for discriminating high vs. low levels of CTGF varied among studies. Also, different cut-off values for age and tumor size were described in various studies, which may cause selection bias for patients. Furthermore, the studies included in the meta-analysis used numerous technologies to evaluate CTGF, including polymerase chain reaction and immunohistochemistry. These methodological discrepancies represent another potential source of bias. Most of the studies included originated from countries in Asia. This is partially due to the high incidence of digestive system cancer in Asian countries [40]. Finally, some of the studies included failed to provide direct data; some of the HRs and 95% CI were calculated from the Kaplan-Meier survival curve. This factor represents another potential source of bias.

Despite the limitations described above, this meta-analysis used data from 1730 patients to evaluate the impact of CTGF expression on clinical characteristics and survival outcomes in patients with digestive system cancer. Our results indicate that increased levels of CTGF expression are significantly associated with an increase in tumor size, multiple tumors, vascular invasion, and advanced patient age. Subgroup analysis by cancer type revealed that increased levels of CTGF were associated with increased risk for advanced TNM staging and lymph node metastasis in patients with GC and decreased risk of TNM staging and lymph node metastasis in patients with CRC. In patients with HCC or GC, increased CTGF levels were associated with poorer OS. In CRC patients, high CTGF expression was associated with improved survival.

## 5. Conclusion

Although pooled results showed no association between CTGF expression and survival, CTGF may nonetheless serve as a novel biomarker for disease status and predicted survival in some types of digestive system cancer. Additional studies will be necessary to validate the findings in this study and the conclusion that CTGF is a valuable biomarker for clinical status in patients with some specific digestive system cancers.

## Figures and Tables

**Table 1:** Characteristics of studies included in the meta-analysis.

First author and year	Region	Tumor type	Sample size	Cut-off value	Detection method	Outcome measures	Variance analysis	Clinical parameters
Kang 2018	China (HK)	GC	145	>50%	IHC	OS	U, M	Gender, age, grade, LNM, TNM stage
Jia 2017	China	HCC	374	NR	IHC	OS, DFS	U, M	Gender, age, size, number, VI, CF
Wang 2010	China	HCC	88	Median	Elisa	OS, DFS	M	Gender, age, size, number, CF
Lin 2005	China	CRC	119	>50%	IHC	OS, DFS	U	Gender, grade, LNM, TNM stage
Kim 2014 (cohort 1)	Korea	HCC	314	>50%	IHC	DFS	U, M	Gender, age, size, grade, TNM stage, number, VI, CF
Kim 2014 (cohort 2)	Korea	HCC	42	>50%	IHC	DFS	NR	Gender, age, size, grade, TNM stage, number, VI, CF
Liu 2008	China	GC	122	>50%	IHC	OS	M	Gender, age, size, grade, LNM, TNM stage
Mao 2010	China	GC	73	Fold change	qRT-PCR	NR	NR	Gender, age, size, grade, LNM, TNM stage
Lin 2011	China (TW)	CRC	136	>50%	IHC	OS, DFS	NR	Gender, size, grade, LNM, TNM stage
Gardini 2005	Italy	ICC	55	>50%	IHC	OS, DFS	U	Grade
Zhou 2009	China	ESCC	80	>25%	IHC	OS	M	Gender, age, grade, LNM, TNM stage
Alvarez 2014	Chile	GBC	182	>50%	IHC	OS	NR	NR

GC: gastric cancer; HCC: hepatocellular carcinoma; CRC: colorectal cancer; ICC: intrahepatic cholangiocarcinoma; ESCC: esophageal squamous cell carcinoma; GBC: gallbladder cancer; IHC: immunohistochemistry; qRT-PCR: quantitative real-time polymerase chain reaction; OS: overall survival; DFS: disease-free survival; RFS: recurrence-free survival; M: multivariate; U: univariate; NR: not reported; LNM: lymph node metastasis; TNM: tumor node metastasis; VI: vascular invasion; CF: capsule formation.

**Table 2:** Meta-analysis results of the association of increased CTGF expression with clinical characteristics.

Clinical characteristics	Studies (*n*)	Number of patients	OR (95% CI)	*P* value	Heterogeneity		
					*I*_2_ (%)	*P*_*h*_	Model
Age (elderly vs. nonelderly)	7	1150	1.38 (1.06, 1.79)	0.02	0%	0.99	Fixed effects
Gender (female vs. male)	9	1405	0.98 (0.76, 1.27)	0.88	32%	0.16	Fixed effects
Tumor size (large vs. small)	5	925	1.40 (1.01, 1.93)	0.04	0%	0.52	Fixed effects
Lymph node metastasis (+ vs. -)	6	675	1.29 (0.61, 2.76)	0.51	78%	0.0003	Random effects
Differentiation (poor vs. well & moderate)	9	1085	0.93 (0.51, 1.70)	0.82	69%	0.001	Random effects
TNM stage (III+IV vs. I+II)	8	1031	1.42 (0.88, 2.28)	0.15	53%	0.04	Random effects
Tumor number (multiple vs. single)	3	730	2.19 (1.23, 3.90)	0.007	0%	0.49	Fixed effects
Vascular invasion (present vs. absent)	3	730	1.46 (1.03, 2.08)	0.04	0%	0.72	Fixed effects
Capsule formation (present vs. absent)	3	730	0.74 (0.24, 2.24)	0.59	79%	0.009	Random effects

**Table 3:** Meta-analysis of the pooled HRs of OS and DFS of different types of cancer with elevated CTGF expression.

Categories	Studies	Number of patients	HR (95% CI)	*P* value	Heterogeneity	
					*I* ^2^ (%)	*P*_*h*_
OS	9	1301	1.05 (0.50, 2.23)	0.89	89%	<0.0001
HCC	2	462	3.60 (1.90, 6.81)	<0.0001	0%	0.95
GC	2	267	1.69 (1.12, 2.55)	0.01	0%	0.80
CRC	2	255	0.17 (0.09, 0.33)	<0.0001	0%	0.49
DFS	7	1127	0.84 (0.37, 1.88)	0.67	87%	<0.0001
HCC	3	444	1.90 (1.38, 2.62)	<0.0001	0%	0.68
Non-HCC	3	310	0.26 (0.16, 0.43)	<0.0001	45%	0.16

## Data Availability

All data relevant to the study are included in the article and uploaded as supplementary information.

## Abbreviations

CTGF: Connective tissue growth factor
TNM: Tumor node metastasis
CCN2: CCN family member 2
Hcs24: Hypertrophic chondrocyte-specific gene product 24
IGFBP-8: Insulin-like growth factor-binding protein 8
HCC: Hepatocellular carcinoma
GC: Gastric cancer
CRC: Colorectal cancer
ICC: Intrahepatic cholangiocarcinoma
ESCC: Esophageal squamous cell carcinoma
GBC: Gallbladder cancer
HRs: Hazard ratios
CI: Confidence intervals
NOS: Newcastle-Ottawa scale
OS: Overall survival
DFS: Disease-free survival.

## Supplementary Materials

Supplementary Materials Suppl Table 1: association between CTGF expression and clinic-pathological features of cancers. Suppl. Figure 1: meta-analysis of the relationship between elevated levels of CTGF and clinical characteristics: (a) gender, (b) lymph node metastasis, (c) tumor differentiation, and (d) TNM stage. Suppl. Figure 2 meta-analysis of the association between elevated levels of CTGF and tumor differentiation, stratified by cancer type. Suppl. Figure 3: meta-analysis of pooled HRs for elevated levels of CTGF. Combined HRs are presented for (a) OS and (b) DFS. Suppl. Figure 4: meta-analysis of combined HRs by OS with increased CTGF expression stratified by sample size (a), country (b), and technology (c).
