# Supplementary material for: Connective Tissue Growth Factor in Digestive System Cancers: A Review and Meta-Analysis
Source: Biomed Res Int. 2020 Dec 27;2020:8489093. doi: 10.1155/2020/8489093 (PMC7781715; doi:10.1155/2020/8489093)
Supplement: Supplementary Materials — Suppl Table 1: association between CTGF expression and clinic-pathological features of cancers. Suppl. Figure 1: meta-analysis of the relationship between elevated levels of CTGF and clinical characteristics: (a) gender, (b) lymph node metastasis, (c) tumor differentiation, and (d) TNM stage. Suppl. Figure 2 meta-analysis of the association between elevated levels of CTGF and tumor differentiation, stratified by cancer type. Suppl. Figure 3: meta-analysis of pooled HRs for elevated levels of CTGF. Combined HRs are presented for (a) OS and (b) DFS. Suppl. Figure 4: meta-analysis of combined HRs by OS with increased CTGF expression stratified by sample size (a), country (b), and technology (c). [file 8489093.f1.pdf]

Supplementary materials

Suppl Table 1. Association between CTGF expression and clinico-pathological features of cancers

Characteristics	Tumor size (cm) (large vs. small)				Lymph node metastasis (positive vs. negative)				Differentiation (poor vs. well, moderate)				TNM Stage (III +IV vs. I + II)				Tumor Number (multiple vs. single)				Vascular Invasion (present vs. absent)				Capsule Formation (present vs. absent)			
Study ID	a1	a0	b1	b0	a1	a0	b1	b0	a1	a0	b1	b0	a1	a0	b1	b0	a1	a0	b1	b0	a1	a0	b1	b0	a1	a0	b1	b0
Kang 2018	-	-	-	-	105	15	19	6	64	56	16	9	93	27	14	11	-	-	-	-	-	-	-	-	-	-	-	-
Jia 2017	42	137	38	157	-	-	-	-	-	-	-	-	-	-	-	-	13	166	5	190	62	117	48	147	93	86	80	115
Lin 2005	-	-	-	-	26	40	31	22	4	62	3	49	32	34	36	17	-	-	-	-	-	-	-	-	-	-	-	-
Kim 2014 (cohort 1)	12	36	52	214	-	-	-	-	29	19	130	136	5	43	12	254	11	37	41	225	29	19	150	116	35	13	225	41
Kim 2014 (cohort 2)	10	7	6	19					10	7	11	14	2	15	1	24	3	14	1	24	13	4	17	8	15	2	25	0
Liu 2009	33	25	33	31	48	10	42	22	26	32	45	19	42	16	38	26	-	-	-	-	-	-	-	-	-	-	-	-
Mao 2010	29	10	23	11	31	8	18	16	29	10	15	19	25	14	18	16	-	-	-	-	-	-	-	-	-	-	-	-
Lin 2011	-	-	-	-	29	32	51	24	5	56	21	54	34	27	55	20	-	-	-	-	-	-	-	-	-	-	-	-
Gardini 2005	-	-	-	-	-	-	-	-	20	13	13	9	-	-	-	-	-	-	-	-		-	-	-	-	-	-	-
Zhou 2009	-	-	-	-	18	21	14	27	4	35	4	37	20	19	15	26	-	-	-	-	-	-	-	-	-	-	-	-

a1, a0 (ref): CTGF overexpression; b1, b0 (ref): normal/low expression of CTGF.

a

Study or Subgroup	high expression Events	high expression Total	low expression Events	low expression Total	Weight	Odds Ratio M-H, Fixed, 95% CI
Jia 2017	29	179	39	195	26.5%	0.77 [0.46, 1.31]
Kang 2018	45	120	9	25	7.9%	1.07 [0.44, 2.61]
Kim 2014 (cohort 1)	12	48	48	266	9.3%	1.51 [0.73, 3.12]
Kim 2014 (cohort 2)	5	17	8	25	3.9%	0.89 [0.23, 3.38]
Lin 2005	28	66	30	53	16.2%	0.56 [0.27, 1.17]
Lin 2011	26	61	42	75	18.3%	0.58 [0.30, 1.15]
Liu 2008	19	58	15	64	8.1%	1.59 [0.72, 3.53]
Mao 2010	17	39	11	34	5.6%	1.62 [0.62, 4.21]
Zhou 2009	14	39	8	41	4.2%	2.31 [0.84, 6.36]
**Total (95% CI)**		**627**		**778**	**100.0%**	**0.98 [0.76, 1.27]**
Total events	195		210			

Heterogeneity: Chi² = 11.84, df = 8 (P = 0.16); I² = 32%
Test for overall effect: Z = 0.15 (P = 0.88)

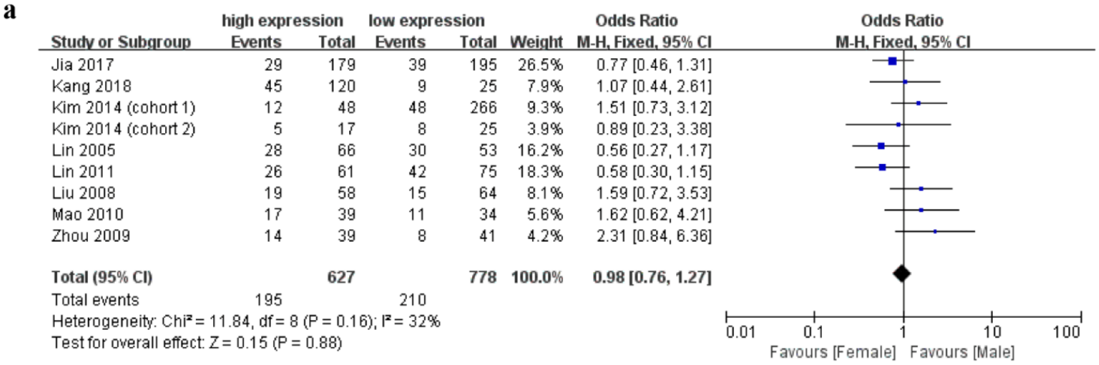

b

Study or Subgroup	high expression Events	high expression Total	low expression Events	low expression Total	Weight	Odds Ratio M-H, Random, 95% CI
Kang 2018	105	120	19	25	15.1%	2.21 [0.76, 6.42]
Lin 2005	26	66	31	53	17.9%	0.46 [0.22, 0.96]
Lin 2011	29	61	51	75	18.2%	0.43 [0.21, 0.86]
Liu 2008	48	58	42	64	16.9%	2.51 [1.07, 5.91]
Mao 2010	31	39	18	34	15.4%	3.44 [1.23, 9.63]
Zhou 2009	18	39	14	41	16.5%	1.65 [0.67, 4.07]
**Total (95% CI)**		**383**		**292**	**100.0%**	**1.29 [0.61, 2.76]**
Total events	257		175			

Heterogeneity: Tau² = 0.70; Chi² = 23.16, df = 5 (P = 0.0003); I² = 78%
Test for overall effect: Z = 0.66 (P = 0.51)

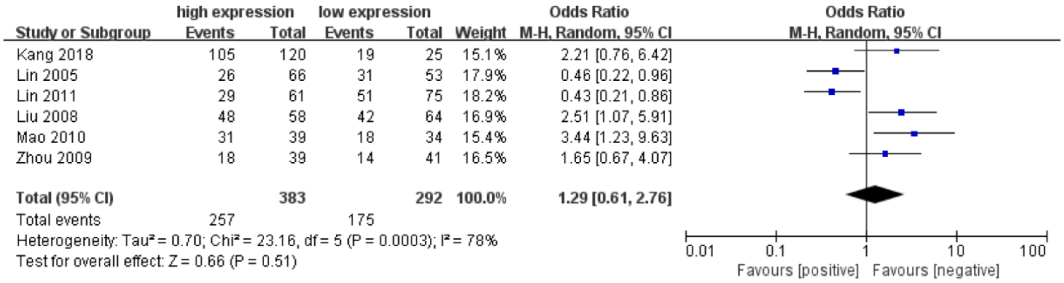

c

Study or Subgroup	high expression Events	high expression Total	low expression Events	low expression Total	Weight	Odds Ratio M-H, Random, 95% CI
Gardini 2005	20	33	13	22	10.8%	1.07 [0.35, 3.20]
Kang 2018	64	120	16	25	12.3%	0.64 [0.26, 1.57]
Kim 2014 (cohort 1)	29	48	130	266	14.3%	1.60 [0.85, 2.99]
Kim 2014 (cohort 2)	10	17	11	25	9.8%	1.82 [0.52, 6.33]
Lin 2005	4	66	3	53	8.0%	1.08 [0.23, 5.03]
Lin 2011	5	61	21	75	11.2%	0.23 [0.08, 0.65]
Liu 2008	26	58	45	64	13.4%	0.34 [0.16, 0.72]
Mao 2010	29	39	15	34	11.6%	3.67 [1.37, 9.86]
Zhou 2009	4	39	4	41	8.5%	1.06 [0.25, 4.56]
**Total (95% CI)**		**481**		**605**	**100.0%**	**0.93 [0.51, 1.70]**
Total events	191		258			

Heterogeneity: Tau² = 0.56; Chi² = 25.98, df = 8 (P = 0.001); I² = 69%
Test for overall effect: Z = 0.23 (P = 0.82)

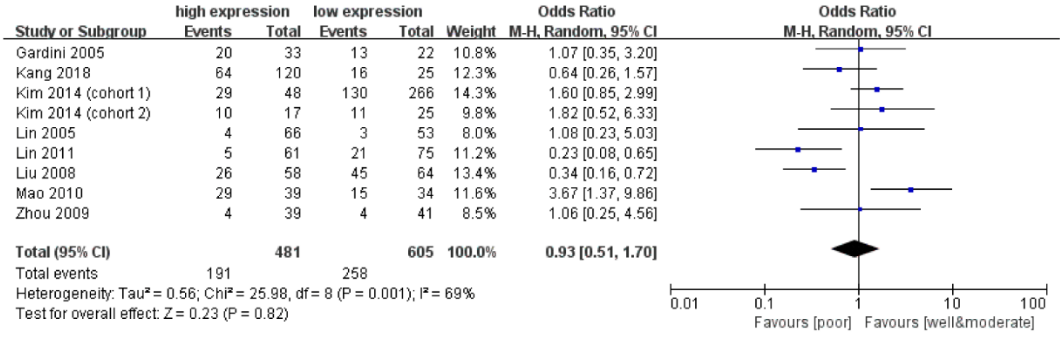

d

Study or Subgroup	high expression Events	high expression Total	low expression Events	low expression Total	Weight	Odds Ratio M-H, Random, 95% CI
Kang 2018	93	120	14	25	13.3%	2.71 [1.10, 6.65]
Kim 2014 (cohort 1)	5	48	12	266	11.5%	2.46 [0.83, 7.34]
Kim 2014 (cohort 2)	2	17	1	25	4.2%	3.20 [0.27, 38.43]
Lin 2005	32	66	36	53	14.8%	0.44 [0.21, 0.94]
Lin 2011	34	61	55	75	15.1%	0.46 [0.22, 0.94]
Liu 2008	42	58	38	64	14.7%	1.80 [0.84, 3.85]
Mao 2010	25	39	18	34	12.9%	1.59 [0.62, 4.06]
Zhou 2009	20	39	15	41	13.4%	1.82 [0.75, 4.46]
**Total (95% CI)**		**448**		**583**	**100.0%**	**1.32 [0.74, 2.34]**
Total events	253		189			

Heterogeneity: Tau² = 0.43; Chi² = 21.26, df = 7 (P = 0.003); I² = 67%
Test for overall effect: Z = 0.94 (P = 0.35)

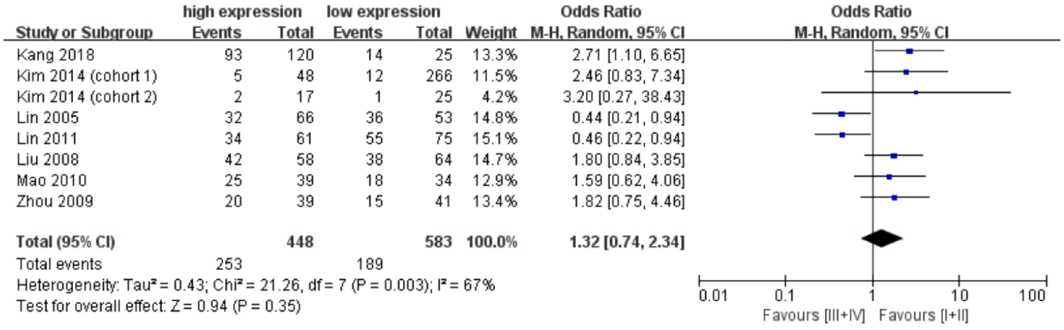

Suppl. Figure 1

Study or Subgroup	high expression Events	high expression Total	low expression Events	low expression Total	Weight	Odds Ratio M-H, Random, 95% CI
**1.11.1 GC**						
Kang 2018	64	120	16	25	12.3%	0.64 [0.26, 1.57]
Liu 2008	26	58	45	64	13.4%	0.34 [0.16, 0.72]
Mao 2010	29	39	15	34	11.6%	3.67 [1.37, 9.86]
**Subtotal (95% CI)**		**217**		**123**	**37.4%**	**0.90 [0.24, 3.47]**
Total events	119		76			
Heterogeneity: Tau² = 1.21; Chi² = 14.31, df = 2 (P = 0.0008); I² = 86%						
Test for overall effect: Z = 0.15 (P = 0.88)						
**1.11.2 HCC**						
Kim 2014 (cohort 1)	29	48	130	266	14.3%	1.60 [0.85, 2.99]
Kim 2014 (cohort 2)	10	17	11	25	9.8%	1.82 [0.52, 6.33]
**Subtotal (95% CI)**		**65**		**291**	**24.1%**	**1.64 [0.94, 2.87]**
Total events	39		141			
Heterogeneity: Tau² = 0.00; Chi² = 0.03, df = 1 (P = 0.86); I² = 0%						
Test for overall effect: Z = 1.73 (P = 0.08)						
**1.11.3 CRC**						
Lin 2005	4	66	3	53	8.0%	1.08 [0.23, 5.03]
Lin 2011	5	61	21	75	11.2%	0.23 [0.08, 0.65]
**Subtotal (95% CI)**		**127**		**128**	**19.2%**	**0.45 [0.10, 2.00]**
Total events	9		24			
Heterogeneity: Tau² = 0.74; Chi² = 2.65, df = 1 (P = 0.10); I² = 62%						
Test for overall effect: Z = 1.06 (P = 0.29)						
**1.11.4 Others**						
Gardini 2005	20	33	13	22	10.8%	1.07 [0.35, 3.20]
Zhou 2009	4	39	4	41	8.5%	1.06 [0.25, 4.56]
**Subtotal (95% CI)**		**72**		**63**	**19.3%**	**1.06 [0.44, 2.56]**
Total events	24		17			
Heterogeneity: Tau² = 0.00; Chi² = 0.00, df = 1 (P = 0.99); I² = 0%						
Test for overall effect: Z = 0.13 (P = 0.89)						
**Total (95% CI)**		**481**		**605**	**100.0%**	**0.93 [0.51, 1.70]**
Total events	191		258			
Heterogeneity: Tau² = 0.56; Chi² = 25.98, df = 8 (P = 0.001); I² = 69%						
Test for overall effect: Z = 0.23 (P = 0.82)						
Test for subgroup differences: Chi² = 3.07, df = 3 (P = 0.38), I² = 2.2%						

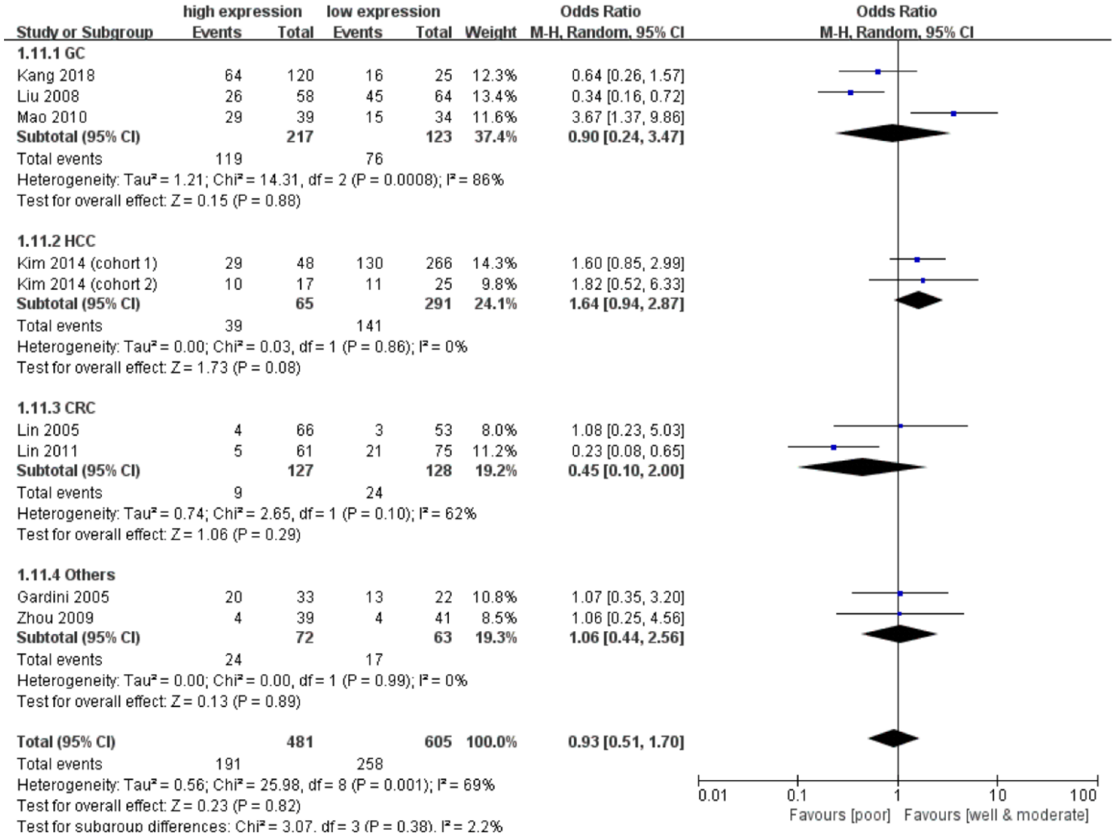

Suppl. Figure 2

a

Study or Subgroup	log[Hazard Ratio]	SE	high expression Total	low expression Total	Weight	Hazard Ratio IV, Random, 95% CI
Alvarez 2014	-0.4277	0.2032	72	110	12.7%	0.65 [0.44, 0.97]
Gardini 2005	-1.1301	0.3493	33	22	11.9%	0.32 [0.16, 0.64]
Jia 2017	1.2988	0.4491	179	195	11.2%	3.66 [1.52, 8.84]
Kang 2018	0.453	0.3436	120	25	11.9%	1.57 [0.80, 3.08]
Lin 2005	-1.9449	0.4316	66	53	11.3%	0.14 [0.06, 0.33]
Lin 2011	-1.5096	0.4696	61	75	11.0%	0.22 [0.09, 0.55]
Liu 2008	0.5653	0.2649	58	64	12.4%	1.76 [1.05, 2.96]
Wang 2010	1.2602	0.4731	59	29	11.0%	3.53 [1.40, 8.91]
Zhou 2009	3.299	1.039	39	41	6.7%	27.09 [3.53, 207.56]
**Total (95% CI)**			**687**	**614**	**100.0%**	**1.05 [0.50, 2.23]**

Heterogeneity: $Tau^2 = 1.10$; $Chi^2 = 76.07$, df = 8 ($P < 0.00001$); $I^2 = 89\%$
Test for overall effect: Z = 0.14 (P = 0.89)

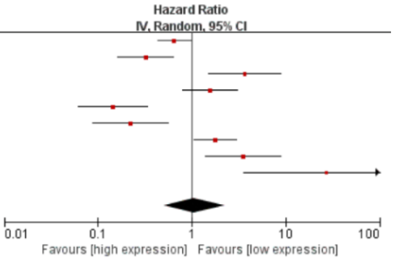

b

Study or Subgroup	log[Hazard Ratio]	SE	high expression Total	low expression Total	Weight	Hazard Ratio IV, Random, 95% CI
Gardini 2005	-0.8052	0.3883	33	22	14.7%	0.45 [0.21, 0.96]
Jia 2017	0.8721	0.4325	179	195	14.3%	2.39 [1.02, 5.58]
Kim 2014 (cohort 1)	0.4453	0.233	48	266	16.1%	1.56 [0.99, 2.46]
Kim 2014 (cohort 2)	0.6841	0.5251	17	25	13.3%	1.98 [0.71, 5.55]
Lin 2005	-1.682	0.395	66	53	14.7%	0.19 [0.09, 0.40]
Lin 2011	-2.0174	0.6804	61	75	11.6%	0.13 [0.04, 0.50]
Wang 2010	0.8696	0.3174	59	29	15.4%	2.39 [1.28, 4.44]
**Total (95% CI)**			**463**	**665**	**100.0%**	**0.84 [0.37, 1.88]**

Heterogeneity: $Tau^2 = 1.01$; $Chi^2 = 47.91$, df = 6 ($P < 0.00001$); $I^2 = 87\%$
Test for overall effect: Z = 0.43 (P = 0.67)

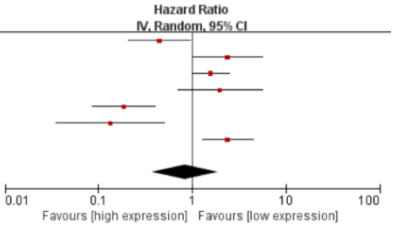

Suppl. Figure 3

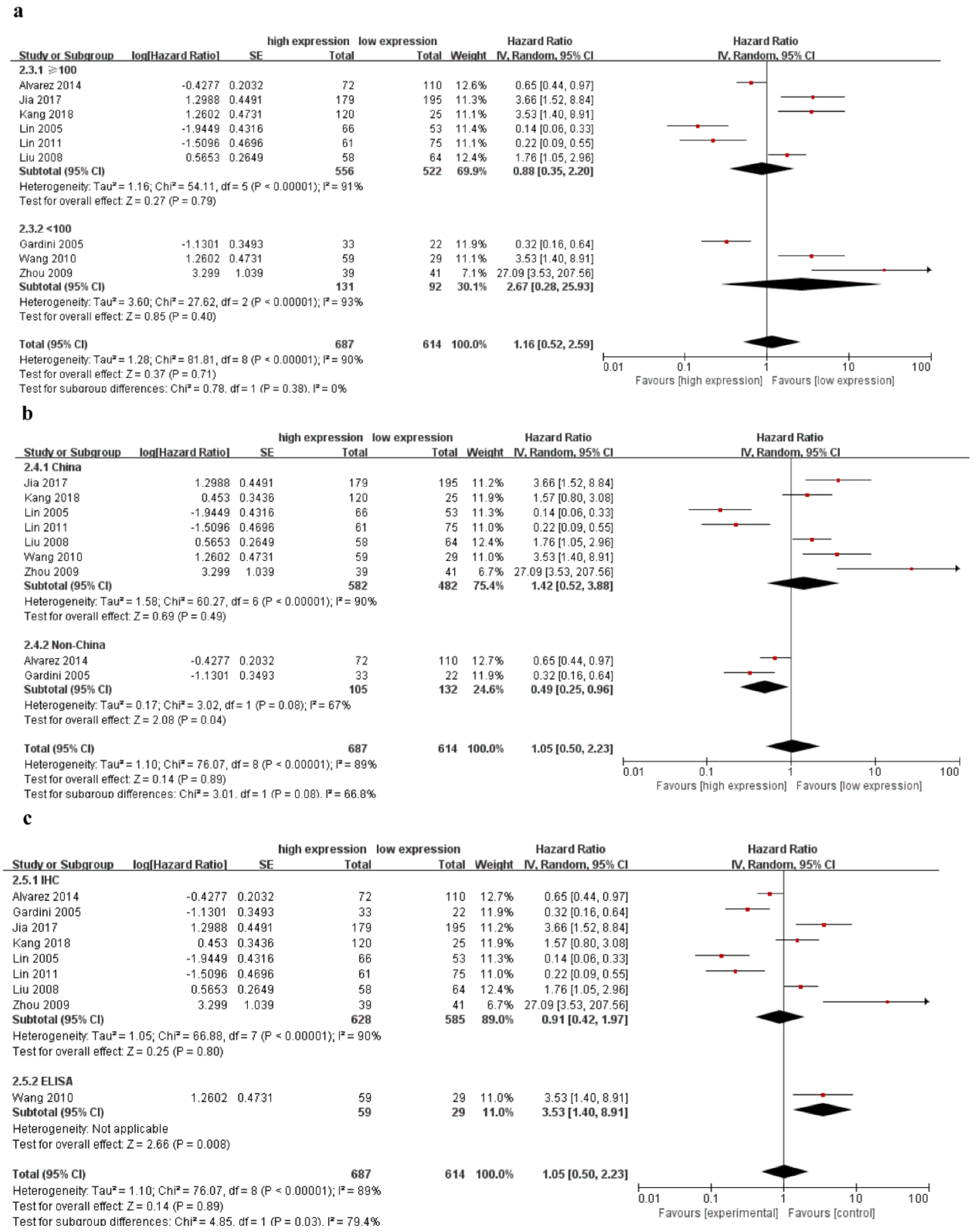

Suppl. Figure 4
